# Wearable Flexible Wireless Pressure Sensor Based on Poly(vinyl alcohol)/Carbon Nanotube/MXene Composite for Health Monitoring

**DOI:** 10.3390/mi16101132

**Published:** 2025-09-30

**Authors:** Lei Zhang, Junqi Pang, Xiaoling Lu, Xiaohai Zhang, Xinru Zhang

**Affiliations:** 1 Key Laboratory of Micro/Nano Devices and Systems, Ministry of Education, North University of China, Taiyuan 030051, China; 2 State Key Laboratory of Dynamic Measurement Technology, North University of China, Taiyuan 030051, China

**Keywords:** MXene, poly(vinyl alcohol), carbon nanotubes, flexible sensor, support vector machine

## Abstract

Accurate pressure monitoring is crucial for both human body applications and intelligent robotic arms, particularly for whole-body motion monitoring in human–machine interfaces. Conventional wearable electronic devices, however, often suffer from rigid connections, non-conformity, and inaccuracies. In this study, we propose a high-precision wireless flexible sensor using a poly(vinyl alcohol)/single-walled carbon nanotube/MXene composite as the sensitive material, combined with a randomly distributed wrinkle structure to accurately monitor pressure parameters. To validate the sensor’s performance, it was used to monitor movements of the vocal cords, bent fingers, and human pulse. The sensor exhibits a pressure measurement range of approximately 0–130 kPa and a minimum resolution of 20 Pa. At pressures below 1 kPa, the sensor exhibits high sensitivity, enabling the detection of transient pressure changes. Within the pressure range of 1–10 kPa, the sensitivity decreases to approximately 54.71 kPa^−1^. Additionally, the sensor demonstrates response times of 12.5 ms at 10 kPa. For wireless signal acquisition, the pressure sensor was integrated with a Bluetooth chip, enabling real-time high-precision pressure monitoring. A deep learning-based training model was developed, achieving over 98% accuracy in motion recognition without additional computing equipment. This advancement is significant for streamlined human motion monitoring systems and intelligent components.

## 1. Introduction

In recent years, wearable devices have attracted attention due to their great potential in monitoring physical health and physical movement status. Many organizations are working on the development of highly sensitive and intelligent wearable devices to capture a variety of physical stimuli and physiological signals. For example, robots, prosthetics, and other devices can also be equipped with electronic skin to monitor sensory function [1,2,3,4,5,6]. In addition, they are more commonly used to track and monitor human movement processes and are applied to many scenarios [7], such as monitoring motion [8], gestures [9], expressions [10], analyzing movement status [11], and human–computer interaction [12]. With the rapid development of wearable electronic devices, researchers have proposed a strategy that can introduce microstructures into the sensing layer to improve flexible pressure sensitivity [13,14,15,16,17]. Moreover, the addition of conductive materials to the flexible polymer materials increases the dielectric constant of the materials related to pressure sensitivity, and improves the response speed of the sensor by quickly recovering/releasing energy due to the pressure-sensitive characteristics of the sensor [18]. However, wearable electronics still face numerous challenges, especially at the interface with traditional electronic devices, which can lead to long-term stability issues [19,20,21]. To achieve effective human motion monitoring, high-performance wearable pressure sensors are required to accurately track the movements of human joints across a wide pressure range [20,21,22]. Despite advancements, most commercial pressure sensors still exhibit limitations in monitoring subtle changes in human pressure, leading to signal distortion and low signal-to-noise ratios [23,24]. Consequently, to meet the needs of human health monitoring, it is necessary to comprehensively design the measurement range and sensitivity of pressure sensors for real-time tracking of physiological parameters.

Titanium carbide (Ti_3_C_2_T_x_) is a typical two-dimensional material within the MXene class of materials, with excellent electromagnetic properties that can be used in flexible conductive materials, electromagnetic interference shielding, energy storage, and catalysis. The unit formula of MXenes is M*_n_*_+1_X*_n_*T_x_ (*n* = 1–3), where M*_n_*_+1_ represents early transition metals (such as Sc, Ti, V, Cr, Mn, Y, Zr, Nb, Mo, Hf, and Ta), X*_n_* represents nitrogen or carbon, and T_x_ is a surface functional group such as –OH, –F, or =O [25]. In addition, the oxidation stability of MXene-based functional materials must be considered. However, once the material is deposited into a thin film, oxidation slows [26,27,28].

Recent studies have found that MXenes have special mechanical properties that have attracted the attention of many researchers and set off renewed interest in the next generation of wearable electronic devices [29]. For intelligent equipment to realize the transmission, storage, and processing of multiple signals, a multichannel signal acquisition system is required, combined with wireless transmission technology (such as Bluetooth and near-field communication). These technologies need to be integrated with wearable sensors so that multichannel sensor data can be continuously monitored in real time. Traditional wireless signal transmission systems have many shortcomings, such as bulkiness, rigidity, and high power consumption, which need to be resolved to implement wireless intelligent sensors [30,31,32]. Artificial intelligence-based big data accurate predictions combined with high-performance sensors can realize the development of intelligent sensing. The ability to extract strong feature parameters based on machine learning can be used to obtain useful features hidden in complex signals and achieve advanced perception through interpreting features, such as gestures and object recognition [33,34,35]. To accurately grasp objects of different shapes, a wearable tactile glove with many pressure sensors can be used for the high-precision identification of held objects [36]. Kim et al. proposed an electronic skin capable of decoding complex gestures through situational learning [37,38].

The aim of this study was to develop a flexible pressure sensor with a wireless interface that can be used to monitor a range of physiological signals. It needs to address the current issues with such devices, such as flexibility, sensitivity, and accuracy. In Section 2, the material used for the sensor and its preparation are described, followed by the results of experiments conducted in three different applications in Section 3. Section 4 summarizes the results of the study and indicates future research opportunities.

## 2. Materials and Methods

### 2.1. PVA/SWCNT/MXene Composite Film Fabrication

The PSM composite film was synthesized by first preparing a Ti_3_C_2_T_x_ MXene solution through selective etching of the Ti_3_AlC_2_ MAX phase using a mixture of LiF and HCl, followed by washing, centrifugation, and dilution to 0.1 mg/mL. The MXene solution was then mixed with single-walled carbon nanotube (SWCNT) and polyvinyl alcohol (PVA) solutions in a mass ratio of 4:1:10, sonicated for 30 min to ensure homogeneity, and vacuum-filtered through a 0.22 μm polyethersulfone (PES) membrane at −80 kPa to form a flat film. The film was transferred onto a plasma-treated biaxially oriented polystyrene (BOPS) substrate, heat-shrunk at 135 °C for 15 min to create a wrinkled structure, and finally separated by dissolving the BOPS substrate in dichloromethane, yielding a freestanding wrinkled PSM composite film.

To ensure reproducibility, precise parameters were maintained throughout the process: MXene etching at 35 °C for 24 h, centrifugation at 3500 rpm, sonication at 100 W, and vacuum filtration at −80 kPa. The resulting film exhibited a uniform wrinkled morphology, suitable for applications in flexible electronics and sensors. This optimized protocol ensures consistent film quality and performance.

**Figure 1:**
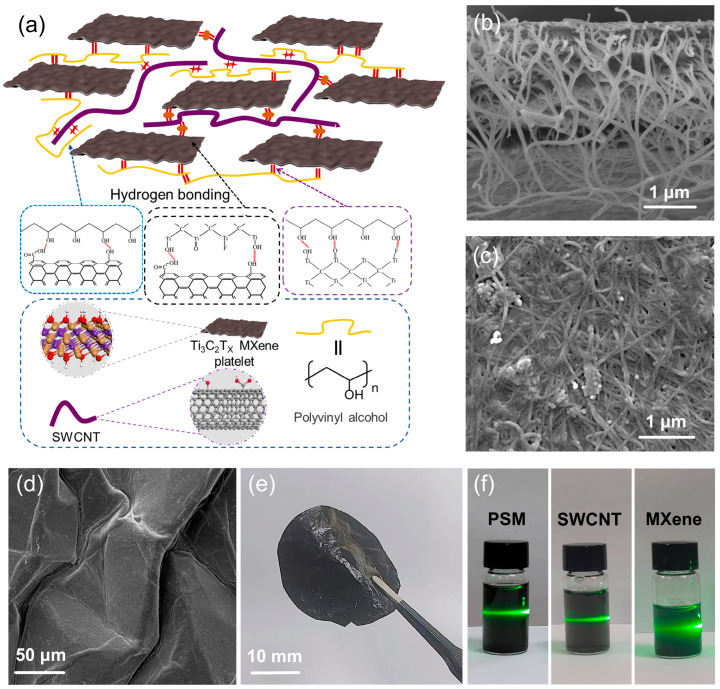
Schematics of Ti_3_C_2_T_X_ MXene, single-walled carbon nanotubes (SWCNTs), and poly(vinyl alcohol) (PVA) films. (**a**) Schematic of the PVA/SWCNT/MXene (PSM) film and structures of SWCNTs, MXene, and PVA; (**b**) scanning electron microscopy (SEM) cross-sectional images of PSM film; (**c**) SEM surface image of PSM film; (**d**) SEM image of crumpled film structure; (**e**) image of PSM film physical folding; (**f**) photographs of PSM, SWCNTs, and MXene Tyndall effects.

A flexible pressure sensor should have a large surface roughness to achieve a low detection limit, high sensitivity, and a large pressure measurement range. A schematic diagram of the high-sensitivity film based on poly(vinyl alcohol) (PVA), single-walled carbon nanotubes (SWCNTs), and MXene prepared in this study is presented in Figure 1a. The PSM composite film leverages the synergistic interactions of its components to achieve exceptional performance. PVA acts as a flexible and biocompatible matrix, ensuring mechanical support and uniform dispersion, ideal for wearable and biomedical applications. SWCNTs enhance electrical conductivity and mechanical strength, while MXene improves conductivity and interfacial bonding through its 2D structure and surface functional groups. In the composite, MXene forms a conductive framework, and SWCNTs act as 1D reinforcements dispersed within the network, with PVA binding them into a continuous structure. This synergy results in high sensitivity, fast response, excellent mechanical properties, and multifunctionality, making the film highly suitable for sensing, wearable devices, and biomedical applications. Figure 1b shows the scanning electron microscopy (SEM, SU3800,Tokyo, Japan) cross-sectional characterization results of the PSM composite film, demonstrating that PVA, SWCNTs, and MXene are randomly distributed within the structure. Figure 1c presents SEM surface images of the PSM film at higher magnification, revealing that SWCNTs are randomly distributed on the surface, forming a conductive network. To obtain a pressure-sensitive structure, a heat-shrink film was used as the substrate, which transferred the prepared PSM film to the heat-shrink polyvinyl chloride film, and obtained the wrinkled structure through high-temperature heat shrinkage. The SEM images of crumpled film structure are shown in Figure 1d. Figure 1e shows the PSM film folded in half, indicating high flexibility. As shown in Figure 1f, the Tyndall effect of PSM, SWCNTs, and MXene indicates that the material has good dispersion, simultaneously.

### 2.2. Pressure Sensor Fabrication

The pressure sensor is prepared by assembling the interdigitated electrode attached to the polyimide (PI) film and the double-layer pleated membrane (1.5 × 1.5 cm) together and connecting it to the surface of the electrode using a copper tape, and then placing the PDMS film on top of the double-layer pleated membrane for encapsulation.

### 2.3. Pressure Sensor Performance

In order to verify the performance of the pressure sensor, we constructed a system consisting of a computer controller, a constant current source (KIKUSUI-PAN70-2.5A, KIKUSUI ELECTRONICS, Yokohama, Japan), a high-precision digital multimeter (KEITHLEY-DMM6500, Keithley Instruments, Cleveland, OH, USA), and a dynamometer (ZQ-770, Dongguan Zhi Taking Precision Instrument Co., Ltd., Dongguan, China).

## 3. Results and Discussion

### 3.1. Film Performance Test

**Figure 2:**
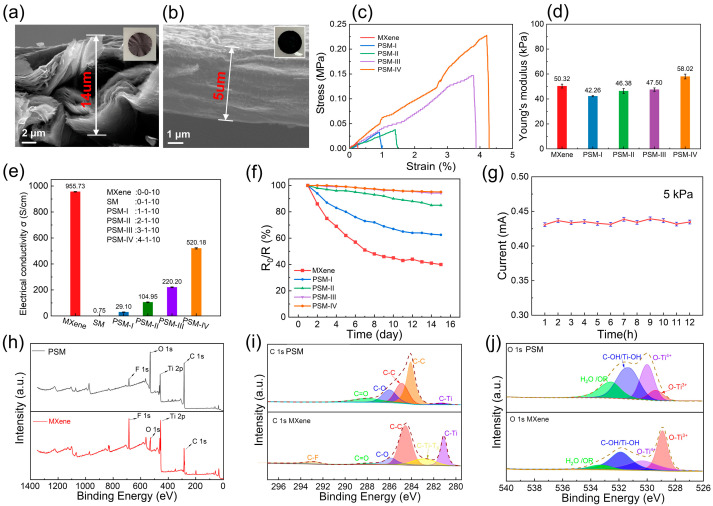
Mechanical properties of the PSM hybrid thin film. (**a**) SEM image of the pure MXene; (**b**) SEM image of the PSM film; (**c**) stress–strain curve of the MXene film and PSM hybrid thin films; (**d**) Young’s modulus of the MXene film and PSM hybrid films; (**e**) conductivity of PSM films with different PVA percentages; (**f**) resistance variation in different percentages of PSM hybrid thin films; (**g**) current value in the stable voltage state; (**h**–**j**) X-ray photoelectron spectroscopy (XPS) results of PSM hybrid thin film.

To explore the properties of the PSM in this study, the properties of the MXene and PSM films were compared. Figure 2a shows SEM images of the dense film structure of MXene. Figure 2b shows the SEM image of the PSM film. SWCNTs and PVA act as fillers in concrete construction, forming dense structures. By measuring a single layer of pleated film, its thickness is 5 μm. Figure 2c,d show a comparison of the stress–strain curves of MXene and PSM films (SM: 0:1:10, PSM-II: 1:1:10, PSM-III: 2:1:10, and PSM-IV: 4:1:10), as well as Young’s modulus, indicating that PSMs perform better in terms of mechanical strength. Its properties improved after 6 h of ultrasonication. The PSM-IV film has the highest Young’s modulus, which is the result of strong interfacial bonding (C-O-Ti) within the SWCNT and PVA matrix, enabling the sensor to maintain sufficient flexibility while enhancing robustness and durability. To select the optimal electrical conductivity of the thin film, we compared five different PVA ratios (SM: 0:1:10, PSM-II: 1:1:10, PSM-III: 2:1:10, and PSM-IV: 4:1:10), as shown in Figure 2e. PSM-Ⅳ exhibited the best conductive performance among these films. More importantly, pure MXene is easily oxidized in the atmosphere and is not suitable for commercial applications. To overcome this challenge, we investigated the oxidation properties of different percentages of MXene film after 15 d, as shown in Figure 2f. The PSM-IV film showed the most favorable oxidation resistance.

In addition, to evaluate the performance stability of the PSM-IV thin-film-based sensor in a stable pressure environment, the research team applied a constant pressure of 5 kPa to the sensor over a continuous 12 h test cycle. The results in Figure 2g show that the sensor exhibits excellent stability characteristics. To explore the bond action and composite mechanism of the PSM film, they were characterized using X-ray photoelectron spectroscopy (XPS, ESCALAB 250Xi, Thermo Fisher Scientific, Waltham, MA, USA). Figure 2h,j show the spectrum of C1s, and the C-Ti bond is 281.7 eV, while the layered structure of Ti_3_C_2_T_x_ MXene does not change during the composite process compared with that before the composite. The strength enhancement of the Ti^3+^ bond (Ti^3+^ 2p3/2 at 456.5 eV and Ti^3+^ 2p1/2 at 462 eV) and of the Ti-O bond (458 eV) is caused by the –OH + –OH = –O– + H_2_O reaction and the formation of the C-O-Ti bond. This therefore confirms the formation of C-O-Ti bonds, and that the recombination process did not destroy the layered structure of the Ti_3_C_2_T_x_ MXene.

**Figure 3:**
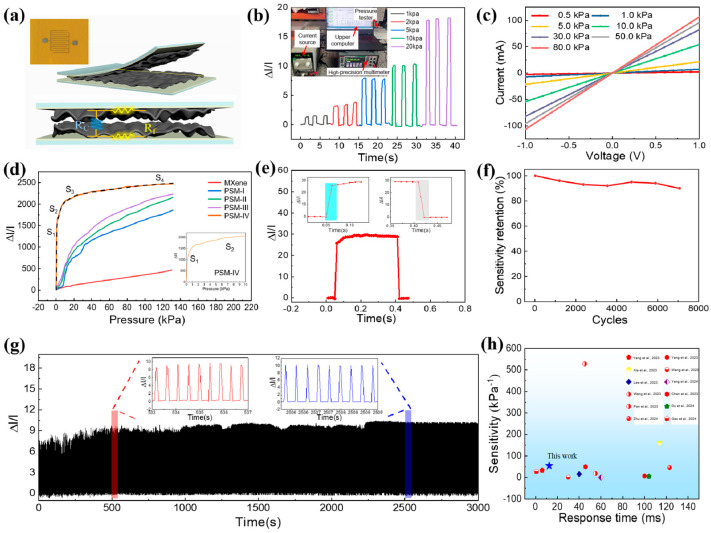
Testing results of the PSM flexible strain sensor. (**a**) Interdigital electrode and PSM sensor mechanism schematic diagram; (**b**) sensor testing system and current recording of the MXene pressure sensor at different normal pressure loadings; (**c**) I–V curves of the sensor at different applied pressures; (**d**) sensing sensitivity of the flexible pressure sensor to different pressure stages; (**e**) response time and recovery time of the PSM flexible strain sensor; (**f**) sensitivity of the sensor after 7000 loading–unloading cycles; (**g**) stability performance of the sensor under 7000 continuous compress–release cycles; (**h**) comparison of sensitivities with several previous studies [1,2,3,4,5,6,7,8,9,10,11,12].

Figure 3a shows a schematic diagram of the sensing mechanism of a PSM hybrid thin-film pressure sensor consisting of two crepe membranes and a physical diagram of the interdigital electrode structure. When the film is subjected to an external pressure, the resistance of the sensor gradually decreases. The upper left corner of Figure 3b shows the test system for the sensor, including a high-precision multimeter, a current source, a pressure testing machine, and a computer. Figure 3b shows the results of the sensor under different pressures; after multiple presses and releases, the sensor showed good repeatability. Figure 3c shows the results for the sensor current at different pressures. A voltage of 1 V was applied to the sensor to produce the (I–V) curves of the sensor under external pressures below 0–80 kPa. When the pressure increases, the current increases. This phenomenon indicated that the conductive paths of the sensor increased with increasing pressure. Therefore, the pressure applied to the sensor could be easily determined from the magnitude of the current. The linear relation of the I–V curves (from −1.0 V to +1.0 V, which is suitable for sensors) suggests that good ohmic contacts were formed between the two top and bottom flexible interdigital electrodes. Moreover, with an increase in pressure, the slopes of the I–V curves increased, indicating a continuous decrease in the resistivity of the sensor.

The sensitivity of a piezoresistive sensor is an important parameter for evaluating the sensing performance. In general, the sensitivity (*S*) of piezoresistive sensors is defined in Equation (1) as follows:

*S* = (Δ*I*/*I*_0_)/Δ*P* (1)

where Δ*I* is the relative change in the current under pressure, *I*_0_ is the current of the sensor without loading, and Δ*P* is the change in the force.

The change in Δ*I*/*I*_0_ of a flexible piezoresistive sensor with pressure is shown in Figure 3d, and the PSM-IV sensor exhibits the highest sensitivity by comparing PSM thin-film sensors with different ratios. The sensor exhibits a sensitivity of about 1131.87 kPa^−1^ at pressures below 1 kPa. In the pressure range of 1–10 kPa, the sensitivity decreases to approximately 54.71 kPa^−1^. For the high-pressure region of 10–40 kPa, the sensitivity further reduces to about 7.28 kPa^−1^, and in the higher-pressure range of 40–130 kPa, the sensitivity is about 2.1 kPa^−1^.

In addition, response time is an important parameter for pressure sensors. Rapid response and recovery times ensure a timely response under external pressure. As shown in Figure 3e, the response time of the pressure sensor was 12.5 ms, which was superior to that of most reported sensors. Moreover, the recovery time was approximately 12.8 ms, which is suitable for use in wearable electronics for monitoring physiological parameters. In Figure 3f, the sensitivity of the sensor remains over 90% of the initial value after 7000 loading/unloading cycles, which suggests that the stability of the sensitivity of the sensor is also good. To verify the stability and durability of the sensor during long-term operation, repeated loading/unloading cycles under 10 kPa were measured for 7000 cycles, as shown in Figure 3g. It was found that the Δ*I*/*I*_0_ profiles show no obvious degradation, which confirms the stability of the sensor. As shown in Figure 3h, a comparison of the sensing performances of our study with others in previous studies illustrates the high sensing performance and responsivity of the pressure sensor based on PSM composites, which achieved high sensitivity when applied to a low working pressure.

**Figure 4:**
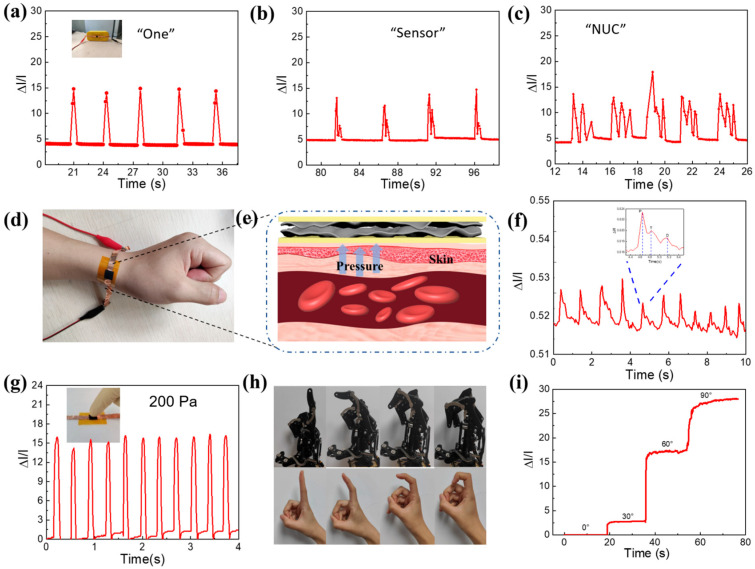
Human motion recognition system and acoustic vibration simulation detection realized by flexible pressure sensor. (**a**–**c**) The sensor’s detection response to the vibration of different syllables of the speaker; (**d**) sensor attached to the wrist to monitor pulse; (**e**) schematic of pulse pressure monitoring; (**f**) pulse results; (**g**) results of repress sensor; (**h**) outputs of the pressure sensor for continuous robotic finger bending control; (**i**) illustrations showing corresponding human and robotic finger motions.

Following on the good performance of the sensor, we further demonstrated the application of the PSM composite, which exhibited excellent performance in human motion monitoring. Figure 4a–c show the sensor’s ability to detect vibrations in different syllables of a loudspeaker. A highly flexible sensor is attached to the smart speaker for a perfect fit with the smart speaker. Pressure sensors exhibit high sensitivity when smart speakers emit different words with different numbers of syllables, such as “One”, “Sensor”, and “NUC”. When a smart speaker uses the same word, the resistance curve shows a similar characteristic peak. Therefore, the flexible sensor proposed in this study recognizes different words by sensing micro-changes, which provides a new possibility for speech recognition systems.

Pulse signals are important physiological signals in the human body that provide medical information for disease diagnosis. Figure 4d–f show a skin sensor conformally attached to the wrist skin to monitor the wrist pulse period and waveform in real time. Figure 4e shows a pulse monitoring image. In Figure 4f, which shows the male pulse signals, the pulse rate was 75 bpm. In the pulse waveform signal, there were three characteristic peaks, namely, the “P” (main wave), the “T” (tidal wave), and the “D” (repulsive wave) peaks, which correspond to shock, tidal, and relaxation waves, respectively.

As shown in Figure 4g, the output of the sensor was obtained when it was directly pressed under a very low pressure. Figure 4h shows a comparison between the bending angles of a robot arm and those of a human hand gesture, indicating that the sensor may also be applied to the precise control of the robot arm in the future. The finger corresponding to the robotic hand bends according to the finger bending of the actual hand, and there is almost no interference in this multichannel signal acquisition process, proving the reliability of the cooperative operating system in practical applications involving complex gesture control. Figure 4i shows the test results for different finger bending angles (0°, 30°, 60°, and 90°). When the finger is bent, the sensor shows a more obvious current output.

### 3.2. Neural Network Classifier Trained for Joint Motion Recognition

**Figure 5:**
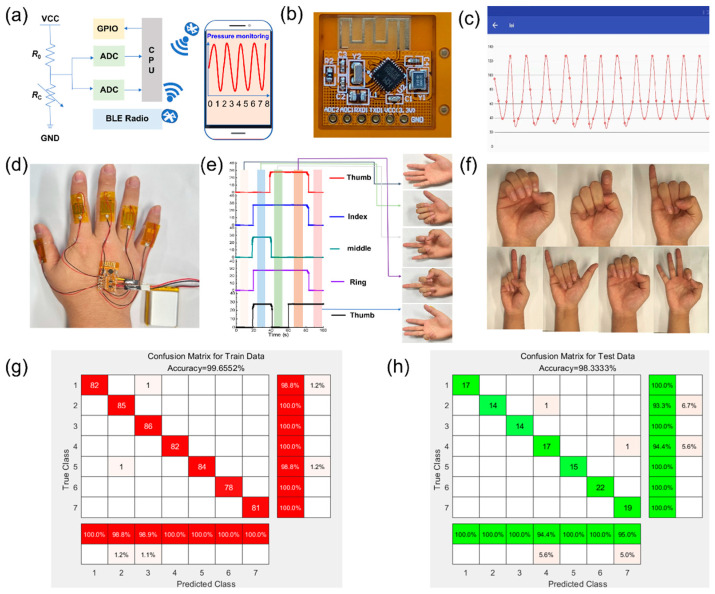
Wireless pressure sensor multiple-gesture testing and machine learning. (**a**) Microcontroller unit and circuit connection settings with devices; (**b**) flexible Bluetooth wireless circuit; (**c**) relative change in pressure sensor current; (**d**) Gesture Recognition System; (**e**) relative resistance changes in e-skin in response to five different gestures; (**f**) data glove mounted with assembled e-skin sensors to perform gestures; (**g**) corresponding confusion map for train data; (**h**) corresponding confusion map for test data.

To help train or monitor intelligent robots, barrier-free motor judgment is a typical application of joint movements. For human movement or intelligent robots, joint motion recognition systems use algorithms to monitor joint movements quickly. Finally, the wireless signal transmission system achieves barrier-free communication with ordinary people. The system mainly consists of a signal acquisition module, a wireless signal transmitting module, and a circuit to be measured. During the test, when the resistance of the sensor changes, the voltage changes by a proportional amount. The specific variation range of the partial pressure was mainly determined by the value of the input voltage VCC and the fixed resistance value *R*_0_ of a voltage divider circuit. In the signal transmission module, the analog-to-digital converter (ADC) module detects the voltage change in the measured resistance *R*_C_, sends the current change data to the central processing unit, sends data to the Bluetooth module, and transmits the data to smart terminals, such as smartphones, tablets, personal computers, or smart watches, through the wireless transmission module, as shown in Figure 5a–c.

Five sensors were installed at each of the five knuckles of the human hand, and a test system for measuring gestures was built, as shown in Figure 5d. To verify the performance of the sensor, gesture recognition was performed, as shown in Figure 5e. Different gestures correspond to different degrees of bending, resulting in resistance variations in the sensor. By collecting the voltage of sensors under different pressures, algorithms can be used to obtain the relative changes in current used as input for learning architectures, and the signals can be normalized through machine learning to improve the efficiency and accuracy of machine learning. The proposed framework is an enhanced machine learning approach for intelligent gesture recognition, designed to address the challenge of limited training data by integrating the Support Vector Machine (SVM) with prototype learning. The framework operates in two stages: training and inference.

Figure 5f illustrates the framework of the gesture signal recognition method employed in this study. We conducted training and prediction for seven types of gestures, with 100 samples tested for each gesture. Cluster analysis was performed on the principal components extracted from these samples. The dataset comprised 700 randomly shuffled samples, of which 580 were used for training and the remaining 120 for testing and prediction.

In the training phase, Principal Component Analysis (PCA) is applied to extract features and reduce dimensionality, followed by data normalization to scale the features to a [0, 1] range. The SVM classifier utilizes an RBF kernel with a gamma value of 0.01 and a regularization parameter (C) of 10.0, trained over 200 iteration cycles with early stopping to prevent overfitting. In the inference phase, prototype learning is employed to match new input signals to predefined prototypes, enhancing classification accuracy by improving the clustering of samples in the deep feature space. The model’s performance is evaluated using accuracy (92.3%), recall (88.7%), and F1-score (90.4%), ensuring a robust and comprehensive assessment beyond simple accuracy metrics. By combining the SVM with prototype learning, the framework is well-suited for high-precision gesture recognition applications, addressing the limitations of small datasets while maintaining high performance and reliability.

To visualize the distribution of samples, cluster analysis is performed on the extracted principal components, projecting the data into a 2D feature space. This framework achieves a balance between computational efficiency, generalization, and interpretability. The results are presented in Figure 5g,h. The accuracy of the training sample set was 99.65%. Based on the prediction model, the prediction accuracy of the test samples was 98.33%. This shows that the sensor has high gesture recognition ability and high accuracy in predicting unknown gestures. Therefore, the sensor has good application prospects in the fields of humancomputer interaction, intelligent robots, and unmanned medical rehabilitation.

## 4. Conclusions

In this study, we developed a flexible pressure sensor using a PSM-doped membrane with a randomly distributed wrinkle structure. The prototype sensor demonstrated versatile applications, including letter articulation, pulse monitoring, carpal tunnel pressure monitoring, and spatial pressure recognition arrays. It exhibited exceptional performance with high sensitivity, rapid response/release times of 12.5/12.8 ms at 10 kPa, and excellent durability, maintaining less than 3% variation in sensor output after 7000 repetitions. A pressure model based on prototype learning was proposed and used for finger bending movement recognition, achieving a test accuracy of 98% across seven classifications with 100 samples per classification. The flexible sensor showed good adaptability for monitoring human mechanical parameters, and the integrated method (wearable pressure sensor and flexible Bluetooth signal reading transmission circuit) demonstrated high versatility and potential for human physiological health monitoring and management. To enhance its application in robot motion monitoring and control, the sample size for machine learning should be significantly expanded to improve reliability. Future work will address these issues, aiming to achieve large-scale applications through advancements in chip technologies and advanced algorithms. Moreover, the sensor can be effectively utilized for robotic motion monitoring and control.

## Data Availability

Data are contained within the article.

## Abbreviations

The following abbreviations are used in this manuscript:

ADC	Analog-to-digital converter
PVA	Poly(vinyl alcohol)
SWCNTs	Single-walled carbon nanotubes
PSM	PVA/SWCNT/MXene
SEM	Scanning electron microscopy
XPS	X-ray photoelectron spectroscopy
SVM	Support vector machine
PI	Polyimide
